# Safety, anti-tumour activity, and pharmacokinetics of fixed-dose SHR-1210, an anti-PD-1 antibody in advanced solid tumours: a dose-escalation, phase 1 study

**DOI:** 10.1038/s41416-018-0100-3

**Published:** 2018-05-14

**Authors:** Hongnan Mo, Jing Huang, Jiachen Xu, Xuelian Chen, Dawei Wu, Dong Qu, Xi Wang, Bo Lan, Xingyuan Wang, Jianping Xu, Honggang Zhang, Yihebali Chi, Qing Yang, Binghe Xu

**Affiliations:** 1 Department of Medical Oncology, National Cancer Center/Cancer Hospital, Chinese Academy of Medical Sciences and Peking Union Medical College, Beijing, 100021 China; 2 Department of Diagnostic Radiology, National Cancer Center/Cancer Hospital, Chinese Academy of Medical Sciences and Peking Union Medical College, Beijing, 100021 China; 3 Jiangsu Hengrui Medicine Co., Ltd., Shanghai, 201100 China

**Keywords:** Cancer immunotherapy, Drug development

## Abstract

**Background:**

To assess the safety profile, pharmacokinetics, pharmacodynamics and preliminary antitumour activity of fixed-dose SHR-1210, a novel anti-PD-1 antibody, in advanced solid tumours.

**Methods:**

A total of 36 patients with advanced solid tumours received intravenous SHR-1210 at 60 mg, 200 mg and 400 mg (4-week interval after first dose followed by a 2-week schedule) until disease progression or intolerable toxicity. The concentration of SHR-1210 was detected for pharmacokinetics, and receptor occupancy on circulating T lymphocytes was assessed for pharmacodynamics.

**Results:**

No dose-limiting toxicities were observed. Maximum administered dose was not reached. Most adverse events were grade 1 or 2. Treatment-related severe adverse events were found in two patients. No treatment-related death was reported. Two complete responses (gastric cancer, bladder carcinoma) and seven partial responses were seen. In responders, the median follow-up time was 16.0 months (range 8.3–19.5), and the median duration of response was not reached (range 2.7–17.5+ months). The half-life of SHR-1210 was 2.94 d, 5.61 d and 11.0 d for 3 dose levels, respectively.

**Conclusions:**

Our results demonstrated a promising antitumour activity and a manageable safety profile of SHR-1210, displayed an explicit PK evidence of the feasibility of fixed dose, and established the foundation for further exploration.

## Introduction

Programmed death-1 (PD-1) expressed by activated T lymphocytes is a pivotal immune checkpoint receptor mediating immunosuppression once binding to the PD-1 ligands PD-L1 and PD-L2 expressed by tumour cells or stromal cells.^1–3^ The inhibition of PD-1 and PD-L1 pathway has emerged as one of the most potential therapeutic strategies in a variety of cancers, such as melanoma, lung cancer, renal cell carcinoma, head and neck squamous cell carcinoma, etc.^4^ Several monoclonal antibodies against PD-1 and PD-L1 have been developed and under development, such as nivolumab, pembrolizumab, atezolizumab, etc., generating remarkable responses in a wide spectrum of cancers.

Monoclonal antibodies are usually given based on the body weight, which has recently been re-evaluated because of the specific properties, and the increased convenience and improved safety of the administration paradigm of fixed dose.^5–7^ The efficacy of pembrolizumab of 200 mg every 3 weeks (Q3W) has been explored in several phase 3 clinical trials,^8–10^ however, with very few pharmacokinetics (PK) evidence of fixed dose. To the best of our knowledge, there are no phase 1 clinical trials evaluating the efficacy of anti-PD-1 antibodies at fixed dose.

Based on this consideration, we initiated this phase 1 trial (NCT02742935) of PD-1 blockade with a novel humanised high-affinity IgG4-kappa monoclonal antibody SHR-1210 in 36 patients with advanced solid tumours, assessing the safety profile, preliminary antitumour activity, PK and receptor occupancy (RO) rate at fixed doses, establishing the feasibility of this dosing strategy and the basis for further clinical expansion.

## Materials and methods

### Patients

Eligible patients had documented advanced solid tumours; an age between 18 and 75 years old; an Eastern Cooperative Oncology Group (ECOG) performance status of 0 or 1 (on a scale from 0 to 5, 0 indicating asymptomatic, and 1 indicating restricted in strenuous activity); had experienced PD or recurrence after at least one systemic treatment for advanced or metastatic disease; at least one measurable lesion according to Response Evaluation Criteria in Solid Tumors (RECIST) (version 1.1); adequate haematologic, hepatic and renal function. Patients with brain metastasis were enrolled only when the lesions had been stable for at least 3 months. Patients with a history of or active autoimmune disease, a concomitant secondary cancer, history of organ transplantation or PD-1/PD-L1 treatments, active hepatitis B or C viral infection, or ongoing systemic immunosuppressive therapy were excluded. Previous cancer treatment, radiotherapy or radiosurgery must have been completed at least 4 weeks before the enrolment. All patients provided written informed consent before the enrolment.

### Study design and procedures

This multicenter, single-arm, open-label, phase 1 clinical trial was approved by the institutional review board and independent ethics committee of the National Cancer Center, Cancer Hospital, Chinese Academy of Medical Sciences. It was conducted in accordance with the Declaration of Helsinki and the international standards of good clinical practice. Informed consents had been obtained from patients.

This study consisted of an initial dose-escalation and subsequent expansion phase. During dose-escalation, patients were treated with SHR-1210 at a fixed dose of 60 mg every 2 weeks, with escalation to 200 and 400 mg. We chose these dose levels mainly based on the PK and toxicity properties of SHR-1210 given in previous phase 1 study conducted in Australia in 2015. Meanwhile, 200 mg Q3W of other anti-PD-1 antibodies, such as Pembrolizumab and Nivolumab, have both been confirmed to have promising efficacy and tolerability in several tumours and have been approved in worldwide. Based on these information and significant efficacy signal in the 60 mg cohort, the dose escalation did not proceed beyond 400. A quantity of 400 mg was the preplanned maximum administered dose. The drug was administered as an intravenous infusion at week 1, week 5, and then every 2 weeks. The first cycle (4 weeks, 28 days) was designed for observation of dose-limiting toxicity (DLT), which was defined as ≥grade 2 uveitis, ≥grade 2 interstitial pneumonia, ≥grade 3 non-haematologic and ≥grade 3 haematologic adverse events (AEs) related to study medications occurring during the first cycle. Dose escalation proceeded when 3 patients had completed the safety observation period at a given dose level without any DLT, otherwise 3 extra patients were required at this dose level, and if the 3 extra patients developed DLT, the dose escalation terminated, the dose prior to which was defined as maximum-tolerated dose (MTD). Intra-patient dose escalation was not permitted. A modified definition of DLT was incorporated in the study’s protocol. Delayed DLT such as severe immune-related AEs was also recorded for safety analysis after the first 4 weeks, but not influencing the dose escalation. No escalation was continued after 400 mg group even if no DLT was observed.

On the basis of initial signals of activity, subsequent expansion cohorts of extra 9 patients each were enrolled at 60, 200 and 400 mg. All treatments were to continue until intolerable toxicity, confirmed disease progression, death or withdrawal of consent. Treatment beyond initial disease progression (PD) was allowed in clinically stable patients (exhibiting controlled symptom despite imagological PD, stable performance status and good tolerance of SHR-1210) at the discretion of the investigator.

### Safety analyses

Safety evaluation, including clinical examination and laboratory assessments were conducted for all patients treated with SHR-1210 at baseline and regular intervals. Notably, the laboratory assessments for endocrine function were repeated every 4 weeks, while the liver and kidney function tests repeated every 2 weeks. SHR-1210 administration could be interrupted or delayed for protocol-defined reasons, but dose modification was not permitted. The severity of all AEs was graded according to the National Cancer Institute Common Terminology Criteria for Adverse Events (NCI CTCAE), version 4.03. General AEs were handled based on established safety guidelines. Specific AEs, such as immune-related AEs were handled according to the study protocol, as well as those clinical routinely used guidelines. Patients could receive the treatment again after the AEs recovered to initial state or grade 1. Severe adverse event (SAE) was defined as any event lead to death, life threaten, hospitalisation or prolonged hospitalisation, forever or severe deformity or dysfunction, innate abnormalities or birth defect and other vital medical events deteriorating the patient’s disease.

### Antitumour activity analyses

Independent radiologic evaluation by CT or MRI was done at baseline and every 8 weeks during the first 6 months, and every 12 weeks thereafter. Overall response rate (ORR) was summarised as the proportion of response-evaluable patients who had a best response of complete response (CR) or partial response (PR), based on RECIST, version 1.1, as assessed by independent radiologists. Disease control rate (DCR) was defined as the proportion of CR, PR and stable disease (SD) patients.

### Pharmacokinetics and pharmacodynamics

The concentration of SHR-1210 was detected for PK studies. Plasma samples for SHR-1210 PK analysis were collected at −0.5 h, 5 min (±2 min), 2 h (±5 min), 6 h (±5 min), 24 h (±5 min), 48 h (±30 min), day 8 (±60 min), day 15 (±60 min), day 22 (±60 min) from the initiation of drug in Cycle 1, and −0.5 h, 5 min (±2 min) from drug administration on day 1 and day 15 from Cycle 2. Samples were stored at −80 °C until measurement. Serum concentrations of SHR-1210 were determined using a validated enzyme-linked immunosorbent assay (ELISA) by Covance (Shanghai) with a lower limit of quantification (LLOQ) of 157 ng/mL. The bioanalytical method validation of the ELISA was performed based on the FDA’s recommendation, including the validation of “Calibration Standards”, “Quality Controls Intra and Inter Assay Bias and Precision”, “Quality Control Samples”, “Selectivity”, etc. Anti-PD-1 antibody was obtained from Sino Biological and anti-human IgG (Fab specific) peroxidase antibody produced in goat was obtained from Sigma-Aldrich. RO on circulating T lymphocytes was assessed for pharmacodynamics studies. The plasma samples for pharmacodynamics studies were also collected at above-mentioned time points expect for 5 min (±2 min) from drug administration on day 1 and 15 from Cycle 2. RO of SHR-1210 was determined as the ratio of CD45+/CD3+ cells after incubation with control IgG4 (in vivo binding sites of SHR-1210) to that observed cells after incubation with SHR-1210 (total available binding sites).

### PD-L1 expression

We measured PD-L1 expression in pretreatment, archival tumour samples with an investigational version of the Human PD-L1 Immunohistochemistry Kit using the 6E8 antibody (Shuwen Biotech Co., Ltd., Zhejiang Province, China). For each sample, the membrane expression of PD-L1 in tumour cells was determined by two independent pathologists blinded to the clinical data. PD-L1 cell scores were generally based on a single section. All the neoplastic cells were scorable. PD-L1 positivity was defined as ≥5% of tumour cell membrane staining.^11^ For cases in which the tissue sample had not been optimally collected or prepared or in which PD-L1 expression could not be assessed, the PD-L1 status was categorised as unevaluable.

### Statistical analyses

All statistical tests for PK and correlative studies analyses used a two-sided significance level 0.05, adjusted for multiple comparisons. The Clopper-Pearson method was used to calculate the 95% CI for ORR. The progression-free survival (PFS) were estimated with the Kaplan–Meier method. PK and pharmacodynamics parameters of SHR-1210 were calculated using non-compartmental approaches by WinNonlin 5.3 software. SPSS statistics version 22 software was used for all analyses.

## Results

### Study patients

A total of 36 patients with advanced solid tumours, including oesophageal squamous cell carcinoma (ESCC), gastric cancer, triple-negative breast cancer (TNBC), colorectal cancer, non-small-cell lung cancer (NSCLC), nasopharyngeal cancer (NPC), hepatocellular cancer, bladder cancer and cervical cancer, were included between April 26, 2016 and December6, 2016 (Table 1). Most patients had been treated with previous chemotherapy, and some of them were heavily-pretreated. All 36 patients were included in the further analysis of safety profiles, clinical activity and PK studies.

**Table 1:** Baseline characteristics

Characteristic	60 mg (*n* = 12)	200 mg (*n* = 12)	400 mg (*n* = 12)	Total (*n* = 36)
*Age, years*				
Median	52	54.5	58.5	56
Range	35–66	35–65	35–65	35–66
*Gender, no. (%)*				
Male	8 (66.7%)	10 (83.3%)	10 (83.3%)	28 (77.8%)
Female	4 (33.3%)	2 (16.7%)	2 (16.7%)	8 (22.2%)
*ECOG PS, no. (%)*				
0	10 (83.3%)	11 (91.7%)	10 (83.3%)	31 (86.1%)
1	2 (16.7%)	1 (8.3%)	2 (16.7%)	5 (13.9%)
*Tumour type, no. (%)*				
Oesophageal squamous cell carcinoma	3 (25.0%)	9 (75.0%)	2 (16.7%)	14 (38.9%)
Gastric cancer	3 (25.0%)	0	2 (16.7%)	5 (13.9%)
Triple-negative breast cancer	2 (16.7%)	1 (8.3%)	1 (8.3%)	4 (11.1%)
Colorectal cancer	0	1 (8.3%)	2 (16.7%)	3 (8.3%)
Non-small-cell lung cancer	2 (16.7%)	0	1 (8.3%)	3 (8.3%)
Nasopharyngeal cancer	2 (16.7%)	0	1 (8.3%)	3 (8.3%)
Hepatocellular carcinoma	0	0	2 (16.7%)	2 (5.6%)
Bladder cancer	0	0	1 (8.3%)	1 (2.8%)
Cervical cancer	0	1 (8.3%)	0	1 (2.8%)
*Previous treatment, no. (%)*				
Surgery	7 (58.3%)	6 (50.0%)	9 (75.0%)	22 (61.1%)
Radiotherapy	7 (58.3%)	8 (66.7%)	3 (25.0%)	18 (50.0%)
Chemotherapy	12 (100.0%)	12 (100.0%)	12 (100.0%)	36 (100.0%)
*Lines of previous chemotherapy, no. (%)*				
1	1 (8.3%)	3 (25.0%)	4 (33.3%)	8 (22.2%)
2	4 (33.3%)	5 (41.6%)	5 (41.6%)	14 (38.9%)
3	3 (25.0%)	2 (16.7%)	2 (16.7%)	7 (19.4%)
4	4 (33.3%)	2 (16.7%)	1 (8.3%)	7 (19.4%)

*ECOG PS* Eastern Cooperative Oncology Group performance status

The data cutoff date was 13 December 2017, with a median follow-up duration of 10.1 months (range 1.0–19.5). The median treatment duration was 3.2 months (range 0.5–19.3), and eight patients remained on study treatment. A total of 28 (77.8%) patients discontinued SHR-1210. The most common reason for treatment discontinuation was disease progression (24/28). Two patients stopped SHR-1210 because of lung infection that may not related to the study treatment. One patient died from upper gastrointestinal haemorrhage, which thought to be related to tumour progression. The other patient stopped SHR-1210 because of grade IV neutropaenia.

### Safety and tolerability

The MTD was not reached, and no DLT (including delayed DLT) was observed in three dose groups. At the date of analysis, 35 patients (97.2%) had experienced at least one AE, and 32 (88.9%) of them were treatment-related AE (TRAE) (Table 2). Most events were grade 1 or 2. Common TRAEs (≥20%) were reactive capillary hemangiomas (30, 83.3%), pruritus (12, 33.3%), and fatigue (11, 30.6%). Notably, most of the patients developed skin capillary hemangioma. The median time to occurrence of capillary hemangioma was 23 days, the severity was mostly grade 1, and no patients terminated SHR-1210 due to this AE. Spontaneous regression of capillary hemangioma could be observed after termination of treatment. TRAEs greater than grade 3 were observed in 4 of 36 patients (11.1%), including grade 3 elevation of creatine phosphokinase MB (CK-MB) in one patient (2.8%); grade 4 neutropaenia, anaemia and thrombocytopenia in one patient (2.8%); grade 3 increased conjugated bilirubin and aspartate aminotransferase in one patient (2.8%); and grade 3 diarrhoea in the other patient (2.8%). No treatment-related death was reported.

**Table 2:** Treatment-related adverse events

Event, no. (%)	60 mg (*N* = 12)			200 mg (*N* = 12)			400 mg(*N* = 12)			Total (*N* = 36)		
	All	Grade 3	Grade 4	All	Grade 3	Grade 4	All	Grade 3	Grade 4	All	Grade 3	Grade 4
Reactive capillary hemangiomas	8 (66.7%)	0	0	10 (83.3%)	0	0	12 (100%)	0	0	30 (83.3%)	0	0
Pruritus	2 (16.7%)	0	0	4 (33.3%)	0	0	6 (50%)	0	0	12 (33.3%)	0	0
Fatigue	5 (41.7%)	0	0	2 (16.7%)	0	0	4 (33.3%)	0	0	11 (30.6%)	0	0
Conjugated bilirubin increased	2 (16.7%)	0	0	4 (33.3%)	0	0	2 (16.7%)	1 (8.3%)	0	8 (22.2%)	1 (2.8%)	0
Rash	3 (25%)	0	0	2 (16.7%)	0	0	2 (16.7%)	0	0	7 (19.4%)	0	0
Blood bilirubin increased	2 (16.7%)	0	0	2 (16.7%)	0	0	2 (16.7%)	0	0	6 (16.7%)	0	0
Alanine aminotransferase increased	2 (16.7%)	0	0	0	0	0	4 (33.3%)	0	0	6 (16.7%)	0	0
Anaemia	3 (25%)	0	0	1 (8.3%)	0	1 (8.3%)	2 (16.7%)	0	0	6 (16.7%)	0	1 (2.8%)
Hypothyroidism	3 (27.3%)^a^	0	0	0^a^	0	0	1 (9.1%)^a^	0	0	4 (12.1%)^a^	0	0
Aspartate aminotransferase increased	3 (25%)	0	0	0	0	0	1 (8.3%)	1 (8.3%)	0	4 (11.1%)	1 (2.8%)	0
Pyrexia	0	0	0	1 (8.3%)	0	0	3 (25%)	0	0	4 (11.1%)	0	0
Blood prolactin increased	3 (25%)	0	0	0	0	0	0	0	0	3 (8.3%)	0	0
Nausea	1 (8.3%)	0	0	0	0	0	2 (16.7%)	0	0	3 (8.3%)	0	0
Neutropaenia	1 (8.3%)	0	0	1 (8.3%)	0	1 (8.3%)	1 (8.3%)	0	0	3 (8.3%)	0	1 (2.8%)
Cortisol increased	1 (8.3%)	0	0	0	0	0	1 (8.3%)	0	0	2 (5.6%)	0	0
Diarrhoea	1 (8.3%)	0	0	0	0	0	1 (8.3%)	1 (8.3%)	0	2 (5.6%)	1 (2.8%)	0
Dizziness	2 (16.7%)	0	0	0	0	0	0	0	0	2 (5.6%)	0	0
Blood CK increased	1 (8.3%)	0	0	1 (8.3%)	0	0	0	0	0	2 (5.6%)	0	0
Proteinuria	0	0	0	1 (8.3%)	0	0	0	0	0	1 (2.8%)	0	0
Hyperthyroidism	0	0	0	1 (8.3%)	0	0	0	0	0	1 (2.8%)	0	0
Abdominal distension	1 (8.3%)	0	0	0	0	0	0	0	0	1 (2.8%)	0	0
Blood CK-MB increased	1 (8.3%)	1 (8.3%)	0	0	0	0	0	0	0	1 (2.8%)	1 (2.8%)	0
Troponin increased	1 (8.3%)	0	0	0	0	0	0	0	0	1 (2.8%)	0	0
Blood myoglobin increased	1 (8.3%)	0	0	0	0	0	0	0	0	1 (2.8%)	0	0
Blood creatinine increased	0	0	0	0	0	0	1 (8.3%)	0	0	1 (2.8%)	0	0
Blood glucose increased	0	0	0	1 (8.3%)	0	0	0	0	0	1 (2.8%)	0	0
Blood growth hormone increased	0	0	0	1 (8.3%)	0	0	0	0	0	1 (2.8%)	0	0
Blood urine present	0	0	0	1 (8.3%)	0	0	0	0	0	1 (2.8%)	0	0
Conjunctivitis	1 (8.3%)	0	0	0	0	0	0	0	0	1 (2.8%)	0	0
Hypersensitivity	1 (8.3%)	0	0	0	0	0	0	0	0	1 (2.8%)	0	0
Influenza like illness	0	0	0	0	0	0	1 (8.3%)	0	0	1 (2.8%)	0	0
Thrombocytopaenia	0	0	0	1 (8.3%)	0	1 (8.3%)	0	0	0	1 (2.8%)	0	1 (2.8%)
Skin hypopigmentation	1 (8.3%)	0	0	0	0	0	0	0	0	1 (2.8%)	0	0
Anorexia	0	0	0	0	0	0	1 (8.3%)	0	0	1 (2.8%)	0	0
Urine discolouration	0	0	0	1 (8.3%)	0	0	0	0	0	1 (2.8%)	0	0

No grade 5 drug-related AEs were reported.

*CK* creatine phosphokinase, *CK-MB* creatine phosphokinase isoenzyme.

^a^Only include patients with normal thyroid function at baseline

Two patients (5.6%) had treatment-related SAE. One patient with cervical cancer (200 mg) developed grade IV neutropaenia and thrombocytopenia, leading to the termination of treatment. The grade 4 neutropaenia appeared 12 days after the first dose of SHR-1210. The results of autoimmune antibodies tests were all negative. Patient refused bone marrow aspiration. After continuous treatment of granulocyte colony-stimulating factor and thrombopoietin, neutropaenia persisted for 3 weeks and patient recovered without any signs of infection. The other patient with ESCC (60 mg) experienced grade 1 elevation of troponin, leading to hospitalisation and suspending of treatment. After coronary angiography excluding the possibility of myocardial infarction, he resumed SHR-1210 and resulted in sustained partial response.

Immune-related AEs (irAEs) were observed in 31 patients (86.1%), the most of which were reactive capillary hemangioma, pruritus, hypo- or hyperthyroidism, abnormal liver function test, diarrhoea and skin rash, etc. Most of the irAEs were grade 1 or 2. The incidence of hypothyroidism in patients who had normal thyroid function at baseline was 12.1% (4/33), which was similar to that of other anti-PD-1 antibodies.^12^ All the patients with hypothyroidism had no symptoms and were successfully treated with replacement therapy. irAEs greater than grade 3 were observed in 2 patients (5.6%): grade 4 neutropaenia, anaemia and thrombocytopenia in one patient (2.8%); and grade 3 diarrhoea in the other patient (2.8%).

### Antitumour activity

**Fig. 1:**
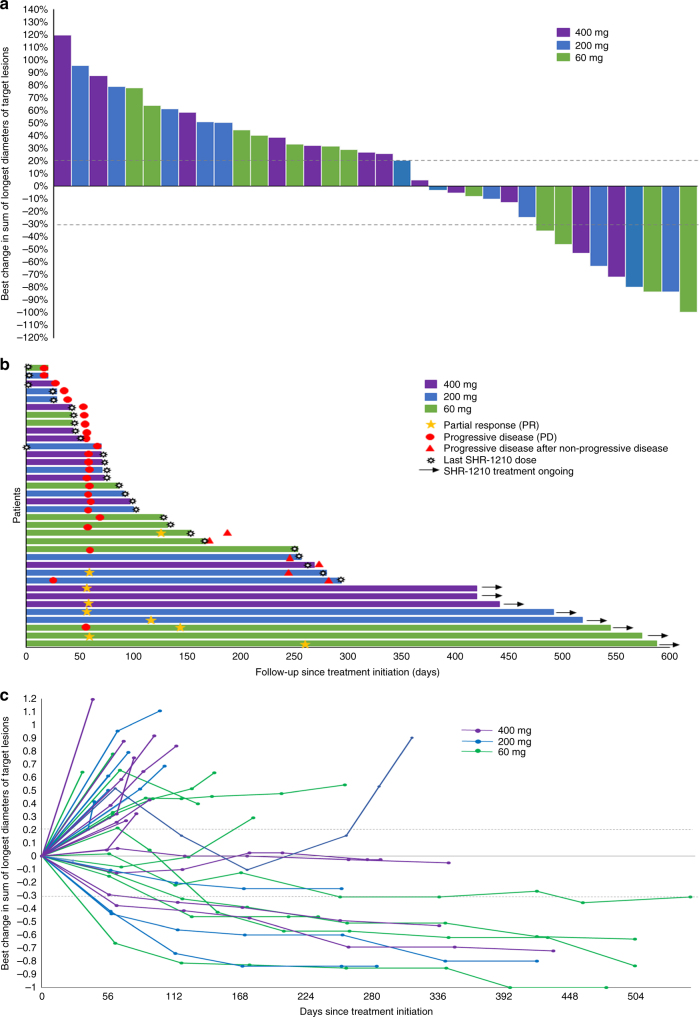
Antitumour activity of SHR-1210 in patients with advanced solid cancers. **a** The best change from baseline in the sum of the longest target lesion diameters per patient. **b** Duration of disease control. **c** Longitudinal change from baseline in the sum of the longest target lesion diameters. Responses were assessed in accordance with the RECIST v1.1 by independent review in all 36 patients. Colour code defines dose level of treatment with SHR-1210. Green, blue, purple bars represent dose levels 60 mg, 200 mg and 400 mg, respectively. The golden pentastar indicates patients with partial response. The red circle indicates patients with progressive disease at the first evaluation. The red triangle indicates patients with progressive disease after non-progressive disease. The black star represents the last dose of SHR-1210 patients receive. The black arrow indicates those patients who are still under treatment at the time of data collection

Based on independent central review assessment, antitumour activity was observed at all doses (Fig. 1). Two patients achieved CR (5.6%, one stomach cancer in 60 mg cohort and one bladder carcinoma in 400 cohort), seven patients achieved PR (19.4%), five patients achieved SD (13.9%), and 22 patients had progressive disease (61.1%). Notably, two patients who were initially categorised as PD had a subsequent partial remission or stable disease in following assessments, and this could represent pseudo-progression. At the date of analysis, objective responses were observed in a substantial proportion of patients with ESCC (3/14, 21.4%), stomach cancer (1/5, 20%), colorectal cancer (1/3, 33.3%), NSCLC (1/3, 33.3%), NPC (1/3, 33.3%), hepatocellular carcinoma (1/2, 50%) and bladder cancer (1/1, 100%), while no response nor disease control were observed in patients with TNBC and cervical cancer. In responders, the median follow-up time was 16.0 months (range 8.3–19.5), and the median duration of response was not reached (range 2.7–17.5+ months).

At the time of the data cutoff, 28 patients (77.8%) had disease progression and 22 patients had died. The median PFS as assessed by independent review and median OS were 1.8 months (95% CI: 1.6–2.0) and 9.8 months (95% CI: 7.3–12.3), respectively. The Kaplan–Meier analysis estimated a 6-month PFS rate of 38.4% (95% CI: 22.3–54.5) and a 6-months OS of 80.6% (95% CI: 67.7–93.5). Among the 28 patients who had progressive disease, progression occurred in preexisting target lesions (10 patients), new metastatic sites (3 patients), or both (15 patients). There are no significant differences among 3 dose groups in terms of PFS or OS. (Supplementary Figure1)

### Pharmacokinetics and pharmacodynamics

**Fig. 2:**
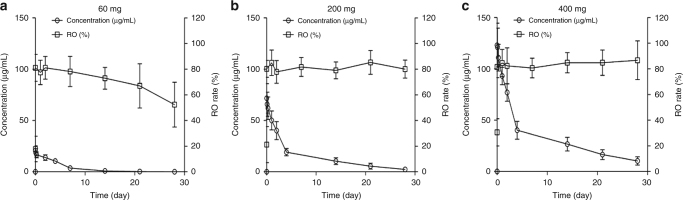
Mean serum concentration-time profiles of SHR-1210 and PD-1 receptor occupancy rates after a single infusion at 60 mg (**a**), 200 mg (**b**) and 400 mg (**c**)

The serum concentration-time profiles of SHR-1210 after a single-intravenous infusion at the dose of 60, 200 and 400 mg are described in Fig. 2. The calculated PK parameters are summarised in Table 3. The mean half-life (*t*_1/2_) of SHR-1210 increased in a dose-dependent manner from 60 to 400 mg, ranging from 2.94 to 11.0 days; similarly, *C*_max_ and AUC were also directly dose-dependent. After repeated doses, the accumulation ratio of SHR-1210 at *C*_min_ from C1D1 to C5D1 was 2.54–3.07 at steady state (1st infusion in Cycle 5); whereas, the accumulation index at the end of infusion (*C*_eoinf_) ranged from 1.08 to 1.53 (Table 4). The current study showed some correlation trends of patients’ body weight with PK properties (Supplementary Figure 2, higher body weight patient tended to have a lower AUC) of SHR-1210, but not with receptor occupancy rate.

**Table 3:** Pharmacokinetic parameters of SHR-1210 following a single infusion at 60 mg, 200 mg and 400 mg

Dose (mg)	60 (*n* = 12)	200 (*n* = 12)	400 (*n* = 12)
*C*_max_ (μg/mL)	20.0 (23.8)	70.4 (19.6)	127 (15.0)
*T*_max_ (days)	0.00347 (0.00347, 0.0347)	0.00347 (0.00347, 0.0833)	0.0833 (0.00347, 2.00)
AUC_0-*t*_ (μg·day/mL)	87.8 (26.7)	437 (26.7)	989 (17.0)
AUC_0-*∞*_ (μg·day/mL)	89.3 (27.0)	465 (26.5)	1160 (20.6)
*t*_1/2_ (days)	2.94 (37.4)	5.61 (23.0)	11.0 (36.6)

Geometric mean (CV%) for *C*_max_, AUC_0-*t*_, AUC_0-*∞*_, and *t*_1/2_; median (range) for *T*_max_.

*n* number of subjects contributing to the mean

**Table 4:** The trough (*C*_min_) and end of infusion (*C*_eoinf_) concentrations of SHR-1210 after the first dose (C1D1) and the dose at steady state (C5D1) and the corresponding accumulation index (AI)

Dose (mg)	Dose regimen	*n*	*C*_min_ (μg/mL) geomean (CV%)	*C*_eoinf_ (μg/mL) geomean (CV%)	AI *C*_min_ (μg/mL) geomean (CV%)	AI *C*_eoinf_ (μg/mL) geomean (CV%)
60	1st infusion in C1	12	0.75 (83.83)^a^	20.01 (22.67)	2.54 (49.60)^b^	1.08 (7.54)
	1st infusion in C5	7	1.48 (124.88)	19.22 (13.34)		
200	1st infusion in C1	12	9.94 (28.32)^c^	70.02 (23.31)	2.56 (12.84)	1.23 (21.46)
	1st infusion in C5	5	29.78 (31.64)	92.66 (31.64)		
400	1st infusion in C1	11	25.84 (26.78)^d^	121.43 (16.66)	3.07 (6.68)	1.53 (13.60)
	1st infusion in C5	5	70.50 (32.23)^e^	184.03 (11.64)		

Only 1 infusion was administered in C1 (4 weeks); Q2W dosing starts from C2.

*AI* accumulation index, calculated as concentration (*C*_min_ or *C*_eoinf_) at C5 divided by the corresponding concentration at C1 of each dose, *C* Cycle, *C*_*min*_day 14 concentration at C1 and C5, *CV%* coefficient of variation %, *n* number of subjects contributing to the mean, *Q2W* every 2 weeks.

^a^*n* = 11

^b^*n* = 6

^c^*n* = 11

^d^*n* = 10

^e^*n* = 4

The PD-1 RO results indicate that SHR-1210 has high affinity to PD-1after a single-intravenous infusion, which is dose-dependent and with a mean peak occupancy of 85% (81, 85 and 88% peak occupancy observed at 60, 200 and 400 mg, respectively) (Fig. 2). Occupancy was durable for at least 28 days after a single infusion at the dose of 200 and 400 mg; while for the 60 mg cohort, there is a trend of occupancy decline to around 50% at the end of day 28. In patients who received repeated Q2W infusions of SHR-1210, occupancy remained high at steady state for the 200 mg and 400 mg cohorts. The occupancy at the trough concentration (*C*_min_) after 1st infusion of Cycle 5 was 67, 77 and 76% for 60, 200 and 400 mg, respectively.

### PD-L1 expression

There were 17 patients for whom tumour biopsy samples were available for PD-L1 assessment. Of these, tumour PD-L1 expression was evaluable in 16 patients. Five patients had PD-L1–positive tumours: two of whom had ESCC and experienced PR, one patient with ESCC had stable disease, the other two patients had disease progression. Among the12 patients with PD-L1–negative tumours, best response was complete response for one patients with gastric cancer, stable disease for two patients (one ESCC and one gastric cancer), and progressive disease for nine patients (six ESCC and two gastric cancer). Notably, the PD-L1 expression of tumour-infiltrating mononuclear cells in the patient with gastric cancer who experienced complete response was relatively high (30%).

## Discussion

To the best of our knowledge, this is the first study reporting the antitumour activity, safety and PK of SHR-1210, a novel anti-PD-1 antibody at a fixed dose in heavily-treated patients with advanced solid tumours, indicating the clinical potential of SHR-1210 due to its promising antitumour activity and a manageable toxicity profile.

The inhibition of PD-1 displays a wide spectrum of clinical antitumour activity among multiple tumours.^4^ The data of this phase I clinical trial demonstrated that 25% of the patients treated with all doses of SHR-1210 had durable objectives responses. The ORR and DCR of ESCC was 21.4 and 42.9%, slightly higher than those treated with nivolumab reported in a phase II clinical trial (17 and 42%, respectively).^13^ The ORR of stomach cancer, lung cancer, NPC, liver cancer, bladder cancer was 20, 33.3, 33.3, 50, 100%, respectively, not completely consistent to the previous reported data,^4^ which may be explained by the limited sample size, yet still providing a comparative clinical efficacy with other checkpoint inhibitors. Notably, no responses or disease control could be observed in triple-negative breast cancer and cervical cancer. In the previously reported clinical trials on immunotherapy in patients with breast cancer, immune checkpoint inhibition like pembrolizumab and atezolizumab demonstrated a response rate of 18.5 and 24%, respectively.^14,15^ In terms of cervical cancer, the ORR of pembrolizumab in PD-L1 positive patients was 17%, and the responses were long-lasting with a mean duration of 26 weeks.^16^ All these results should be interpreted with caution due to the unselected patients regardless of PD-L1 expression and the small sample size in this study.

In addition to the responses, durable objective responses were also noteworthy in multiple types of tumours. Within the median follow-up time of 10.1 months, the median duration of response was not reached. The longest duration has reached 17.5+ months, and the response was still lasting. The relatively long-lasting duration of response was similar with those treated with pembrolizumab and nivolumab.^13,16–18^ Interestingly, two patients experienced PD after treatment of SHR-1210, followed by subsequent remission or stable status after continuous treatment with the immune checkpoint inhibitor. Response after initial progression, which would otherwise be classified as PD by RECIST 1.1, is described as “pseudo-progression”. This a challenging phenomenon during immune checkpoint inhibitor treatment, as several studies had reported,^19,20^ and that is why we need to confirm the response at least 4 weeks after the first evaluation of PD, in order to avoid the premature termination of treatment because of pseudo-progression, offering the necessity of the combination or alteration of response evaluation criteria from conventional RECIST 1.1 to immune-related response criteria (iRECIST).^21^

Most of the toxicities reported in this study were grade 1 or 2, which could be resolved with appropriate supportive treatment, and were consistent with other previously reported anti-PD 1 antibodies,^8,9,13,16–18,22^ suggesting this therapy could be delivered in an outpatient setting. The most commonly reported AE was reactive capillary hemangioma (30, 83.3%), which appeared after the initiation of treatment and regressed spontaneously both during and after treatment. Despite the notably high incidence, no patients discontinued or postponed the treatment due to this AE. Although, immune-related skin events were not unexpectedly, reactive capillary hemangioma is a rare phenomenon and has never been reported in other anti-PD-1 antibodies previously. The mechanism of capillary hemangioma was still under investigation, with a possible explanation of the imbalance between enhancers and inhibitors of angiogenesis as several reports suggested.^23,24^ The investigation of the mechanism of capillary hemangioma induced by SHR-1210 is ongoing.

Another critical question this study brought up is that can anti-PD-1 antibody at fixed-dose reach the comparable clinical efficacy with given based on body weight. Dosing of therapeutic monoclonal antibodies is usually given based on body weight. However, this traditional dosing paradigm has recently been re-evaluated because of the specific properties and the increased convenience, elimination of wastage, improved safety, and improved compliance of fixed dose.^5–7^ A quantity of 200 mg Q3W of pembrolizumab has been confirmed to have comparable efficacy and tolerability in melanoma and NSCLC clinical trials. Based on PK properties of given pembrolizumab either in weight-based or fixed doses, studies demonstrated that both dosing strategies were appropriate.^25^ With a similar maximal efficacy and acceptable tolerability, the administration of pembrolizumab at fixed dose was approved in NSCLC and head and neck squamous cell carcinoma in the United States,^8,10,18,26,27^ and is continuing to be investigated in trials for various indications. For SHR-1210 in patients with advanced solid tumours, our results showed some correlation trends of patients’ body weight with PK properties of SHR-1210, but not with receptor occupancy rate. Meanwhile, the CV% for the exposure of SHR-1210, as shown in Table 3, at different dosing regimens were low (<30%), which indicated that the impact of weight on the drug exposure was limited and none of the changes in SHR-1210 exposure associated with the weight implicated any clinically meaningful differences in safety profile. In addition, previous study of SHR-1210 demonstrated a comparable exposure following a fixed dose (200 mg) and a weight-based dosing (3 mg/kg) (Supplementary Figure 3). In conclusion, with a promising antitumour efficacy and acceptable tolerability, SHR-1210 administered at fixed dose could be utilised in subsequent trials.

The pharmacodynamics of SHR-1210 were assessed according to PD-1 RO on circulating T lymphocytes. The RO is an important factor to test whether the drug develops biological activity by determining whether the pathway leading to antitumour activity is effectively saturated,^28^ and it’s one of the most vital parameters to determine the recommended dose of the drug. Our data display that the drug concentration could be basically maintained higher than 2000 ng/ml to realise sufficient RO saturation within 28 days after the initial infusion at a dose not lower than 200 mg. Based on the above-mentioned studies and analysis, SHR-1210 given at a fixed dose of 200 mg could display a sufficient clinical efficacy and have acceptable tolerance. Although, the frequency of administration requires clinical support from further studies, the fixed dose of 200 mg could be the recommended dose in the further clinical expansion.

Data from several studies suggested improved response of anti-PD-1 antibodies in patients with PD-L1 positive tumours.^22,27,29^ A series of trials have enroled only patients with PD-L1 positive tumours.^10,16,17^ Our data also suggest a possible association between anti-tumour activity and higher PD-L1 expression on tumour cells, although the number of patients is too small to make definite conclusions. It’s premature to assert the validity of PD-L1 as predictive biomarker in patients treated with SHR-1210.

In summary, our results demonstrated a promising antitumour activity and a manageable safety profile of SHR-1210 in pretreated patients with advanced solid tumours, displayed an explicit PK and RO evidence of the feasibility of fixed dose, and determined the recommended dose of 200 mg.

## Electronic supplementary material

Supplementary Figure 1

Supplementary Figure 2

Supplementary Figure 3

## Availability of data and material

The datasets generated during and/or analysed during the current study are available from the corresponding authors on reasonable request.

## References

[1] Dong H (2002). Tumor-associated B7-H1 promotes T-cell apoptosis: a
potential mechanism of immune evasion. Nat. Med..

[2] Dong H, Zhu G, Tamada K, Chen L (1999). B7-H1, a third member of the B7 family, co-stimulates
T-cell proliferation and interleukin-10 secretion. Nat. Med..

[3] Freeman GJ (2000). Engagement of the PD-1 immunoinhibitory receptor by a
novel B7 family member leads to negative regulation of lymphocyte
activation. J. Exp. Med..

[4] Bardhan K, Anagnostou T, Boussiotis VA (2016). The PD1:PD-L1/2 pathway from discovery to clinical
implementation. Front. Immunol..

[5] Bai S (2012). A guide to rational dosing of monoclonal
antibodies. Clin. Pharmacokinet..

[6] Wang DD, Zhang S, Zhao H, Men AY, Parivar K (2009). Fixed dosing versus body size-based dosing of
monoclonal antibodies in adult clinical trials. J. Clin. Pharmacol..

[7] Zhang S, Shi R, Li C, Parivar K, Wang DD (2012). Fixed dosing versus body size-based dosing of
therapeutic peptides and proteins in adults. J. Clin. Pharmacol..

[8] Bauml J (2017). Pembrolizumab for platinum- and cetuximab-refractory
head and neck cancer: results from a single-arm, phase II study. J. Clin. Oncol..

[9] Bellmunt J (2017). Pembrolizumab as second-line therapy for advanced
urothelial carcinoma. N. Eng. J. Med..

[10] Reck M (2016). Pembrolizumab versus chemotherapy for PD-L1-positive
non-small-cell lung cancer. N. Eng. J. Med..

[11] Huang J, Xu B, Mo H, Zhang W, Chen X, Wu D (2018). Safety, activity, and biomarkers of SHR-1210, an
anti-PD-1 antibody, for patients with advanced esophageal
carcinoma. Clin. Cancer Res..

[12] Sznol M (2017). Endocrine-related adverse events associated with
immune checkpoint blockade and expert insights on their
management. Cancer Treat. Rev..

[13] Kudo T (2017). Nivolumab treatment for oesophageal squamous-cell
carcinoma: an open-label, multicentre, phase 2 trial. Lancet Oncol..

[14] Gibson J (2015). Anti-PD-L1 for metastatic triple-negative breast
cancer. Lancet Oncol..

[15] Nanda R (2016). Pembrolizumab in patients with advanced
triple-negative breast cancer: phase I b KEYNOTE-012 study. J. Clin. Oncol..

[16] Frenel JS (2017). Safety and efficacy of pembrolizumab in advanced,
programmed death ligand 1-positive cervical cancer: results from the phase I b
KEYNOTE-028 trial. J. Clin. Oncol..

[17] Muro K (2016). Pembrolizumab for patients with PD-L1-positive
advanced gastric cancer (KEYNOTE-012): a multicentre, open-label, phase 1b
trial. Lancet Oncol..

[18] Seiwert TY (2016). Safety and clinical activity of pembrolizumab for
treatment of recurrent or metastatic squamous cell carcinoma of the head and
neck (KEYNOTE-012): an open-label, multicentre, phase 1b trial. Lancet Oncol..

[19] Nishino M (2012). Personalized tumor response assessment in the era of
molecular medicine: cancer-specific and therapy-specific response criteria to
complement pitfalls of RECIST. Am. J. Roentgenol..

[20] Nishino M, Tirumani SH, Ramaiya NH, Hodi FS (2015). Cancer immunotherapy and immune-related response
assessment: the role of radiologists in the new arena of cancer
treatment. Eur. J. Radiol..

[21] Wolchok JD (2009). Guidelines for the evaluation of immune therapy
activity in solid tumors: immune-related response criteria. Clin. Cancer Res..

[22] Topalian SL (2012). Safety, activity, and immune correlates of anti-PD-1
antibody in cancer. N. Eng. J. Med..

[23] Piraccini BM (2010). Periungual and subungual pyogenic
granuloma. Br. J. Dermatol..

[24] Piguet V, Borradori L (2002). Pyogenic granuloma-like lesions during capecitabine
therapy. Br. J. Dermatol..

[25] Freshwater T (2017). Evaluation of dosing strategy for pembrolizumab for
oncology indications. J. Immunother. Cancer.

[26] Chow LQM (2016). Antitumor activity of pembrolizumab in
biomarker-unselected patients with recurrent and/or metastatic head and neck
squamous cell carcinoma: results from the phase I b KEYNOTE-012 expansion
cohort. J. Clin. Oncol..

[27] Garon EB (2015). Pembrolizumab for the treatment of non-small-cell lung
cancer. N. Eng. J. Med..

[28] Lindauer A (2017). Translational pharmacokinetic/pharmacodynamic modeling
of tumor growth inhibition supports dose-range selection of the anti-PD-1
antibody pembrolizumab. CPT: Pharmacomet. Syst. Pharmacol..

[29] Taube JM (2014). Association of PD-1, PD-1 ligands, and other features
of the tumor immune microenvironment with response to anti-PD-1
therapy. Clin. Cancer Res..

