# Supplementary figures and images for: Safety, anti-tumour activity, and pharmacokinetics of fixed-dose SHR-1210, an anti-PD-1 antibody in advanced solid tumours: a dose-escalation, phase 1 study
Source: Br J Cancer. 2018 May 14;119(5):538–45. doi: 10.1038/s41416-018-0100-3 (PMC6162236; doi:10.1038/s41416-018-0100-3)

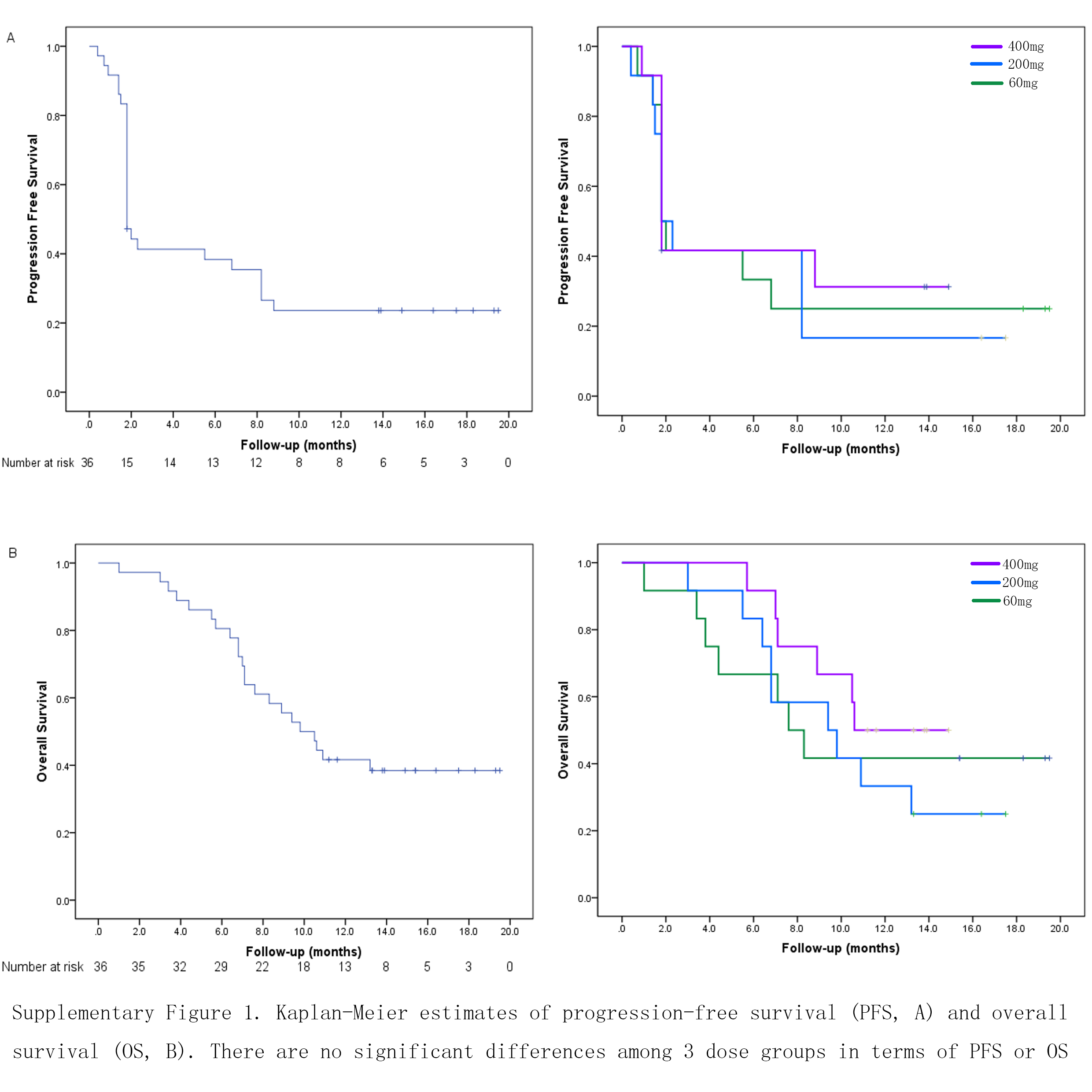

Supplement: Supplementary file 1 — Supplementary Figure 1 [file 41416_2018_100_MOESM1_ESM.tif]

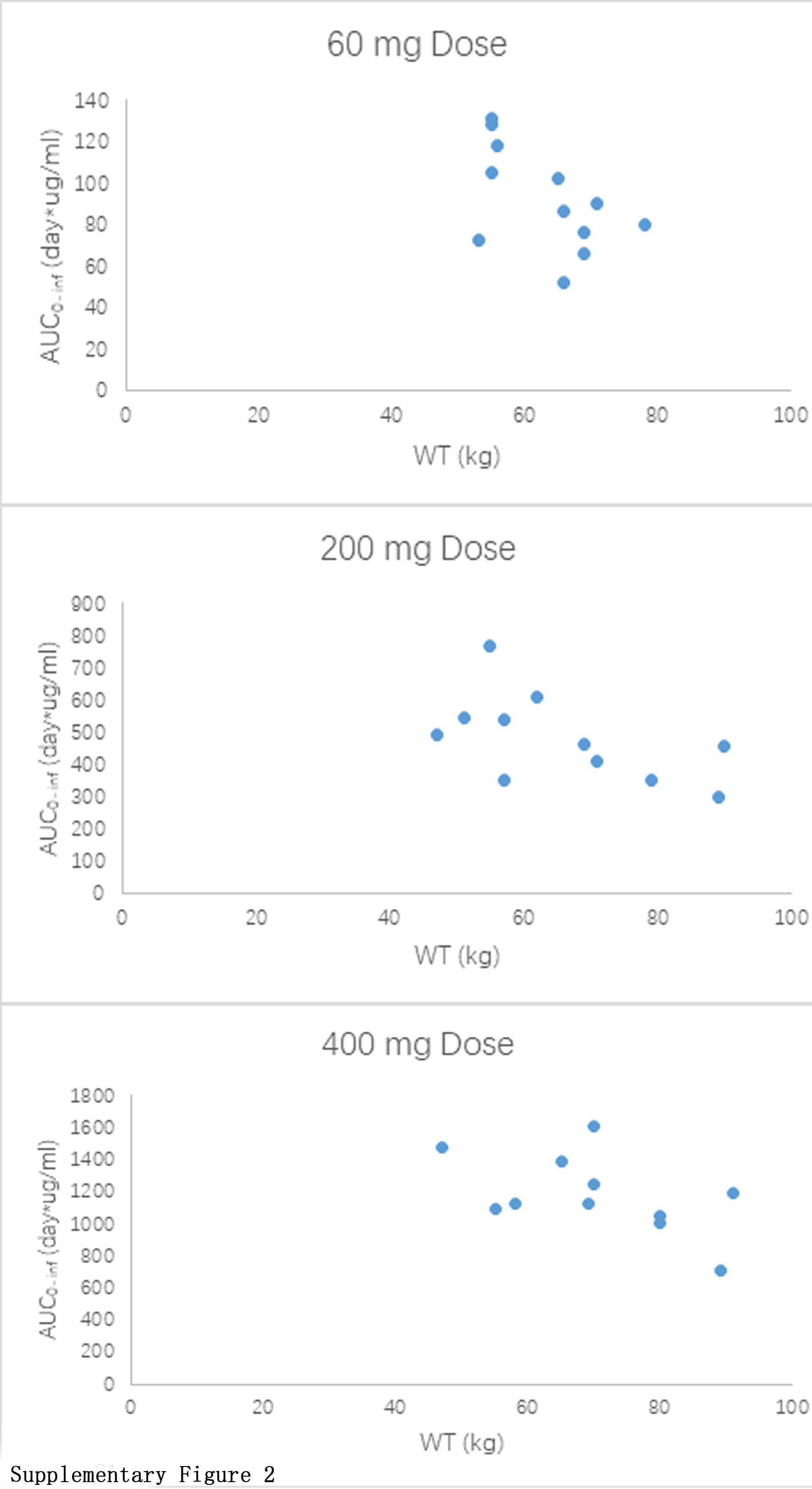

Supplement: Supplementary file 2 — Supplementary Figure 2 [file 41416_2018_100_MOESM2_ESM.tif]

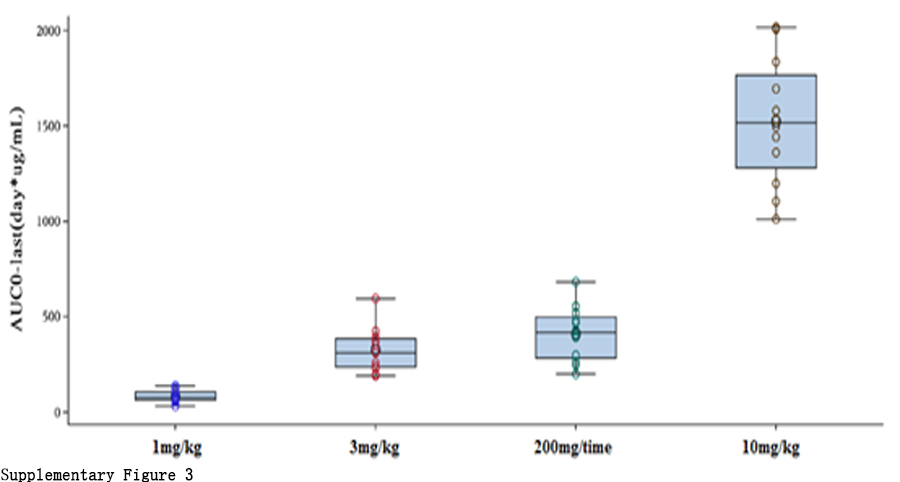

Supplement: Supplementary file 3 — Supplementary Figure 3 [file 41416_2018_100_MOESM3_ESM.tif]
